# Toxicometabolomics of the new psychoactive substances α-PBP and α-PEP studied in HepaRG cell incubates by means of untargeted metabolomics revealed unexpected amino acid adducts

**DOI:** 10.1007/s00204-020-02742-1

**Published:** 2020-04-20

**Authors:** Sascha K. Manier, Lea Wagmann, Veit Flockerzi, Markus R. Meyer

**Affiliations:** 1 Department of Experimental and Clinical Toxicology, Institute of Experimental and Clinical Pharmacology and Toxicology, Center for Molecular Signaling (PZMS), Saarland University, 66421 Homburg, Germany; 2 Department of Experimental and Clinical Pharmacology, Institute of Experimental and Clinical Pharmacology and Toxicology, Center for Molecular Signaling (PZMS), Saarland University, 66421 Homburg, Germany

**Keywords:** Toxicometabolomics, HepaRG, Untargeted metabolomics, HPLC–HRMS/MS, New psychoactive substances

## Abstract

Toxicometabolomics, essentially applying metabolomics to toxicology of endogenous compounds such as drugs of abuse or new psychoactive substances (NPS), can be investigated by using different in vitro models and dedicated metabolomics techniques to enhance the number of relevant findings. The present study aimed to study the toxicometabolomics of the two NPS α-pyrrolidinobutiophenone (1-phenyl-2-(pyrrolidin-1-yl)butan-1-one, α-PBP) and α-pyrrolidinoheptaphenone (1-phenyl-2-(pyrrolidin-1-yl)heptan-1-one, α-PEP, PV8) in HepaRG cell line incubates. Evaluation was performed using reversed-phase and normal-phase liquid chromatography coupled with high-resolution mass spectrometry in positive and negative ionization mode, respectively, to analyze cells and cell media. Statistical evaluation was performed using one-way ANOVA, principal component discriminant function analysis, as well as hierarchical clustering. In general, the analysis of cells did not mainly reveal any features, but the parent compounds of the drugs of abuse. For α-PBP an increase in *N*-methylnicotinamide was found, which may indicate hepatotoxic potential of the substance. After analysis of cell media, significant features led to the identification of several metabolites of both compounds. Amino acid adducts with glycine and alanine were found, and these have not been described in any study before and are likely to appear in vivo. Additionally, significant changes in the metabolism of cholesterol were revealed after incubation with α-PEP. In summary, the application of metabolomics techniques after HepaRG cells exposure to NPS did not lead to an increased number of identified drug metabolites compared to previously published studies, but gave a wider perspective on the physiological effect of the investigated compounds on human liver cells.

**Electronic supplementary material:**

The online version of this article (10.1007/s00204-020-02742-1) contains supplementary material, which is available to authorized users.

## Introduction

Several in vitro models have been developed and used for investigating the metabolism of drugs of abuse (DOA) and new psychoactive substances (NPS), and also for xenobiotics in general (Manier et al. 2018; Richter et al. 2017a, b; Sinz 2012; Sinz and Kim 2006). They are based on subcellular fractions such as pooled human liver microsomes (pHLM), pooled human liver cytosol, or pooled human liver S9 fraction (pHLS9), as well as hepatic cell lines such as HepG2 and HepaRG. Additionally, primary cells can be used after isolation from tissue by enzymatic digestion or mechanical force (Sinz and Kim 2006). Primary human hepatocytes are regarded as the most suitable in vitro model predict in vivo metabolism, since a high number of reactions can be observed such as those catalyzed by cytochrome P450 (CYP) enzymes and by flavin-containing monooxygenases (FMOs), as well as conjugation with glucuronic acid, sulfate, and glutathione (Sinz and Lyubimov 2011). In subcellular fractions, only some of these reactions can be observed. For instance, pHLM are limited to reactions catalyzed by membrane-bound enzymes such as CYP enzymes, FMOs, and uridine 5′-diphospho-glucuronosyltransferases (UGTs). Since these reactions are the most relevant ones in drug metabolism, pHLM are widely applied in such studies (Sinz and Lyubimov 2011). Due to their limitations, pHLM still might fail to predict in vivo excretion products, particularly those that were subject to phase II metabolism other than glucuronidation or a combination of both phase I and II metabolism. In pHLS9, an unseparated mixture of cytosol and microsomes, both phase I and phase II metabolites, are present, but it generally suffers from lower enzyme activities compared to isolated fractions (Brandon et al. 2003; Zhang et al. 2012). Immortalized cell lines such as HepaRG and HepG2 are often applied to avoid the high costs of primary human hepatocytes and to apply a model that is better suited to represent the physiology of liver cells (Sinz and Kim 2006).

Metabolomics studies are dedicated to investigate the change of all detectable small molecules in a biological system on a global or network scale (Liu and Locasale 2017). This includes in silico methods such as automated peak detection and integration, as well as methods from uni- and multivariate statistics to evaluate the results and obtain an impartial image of the changes (Barnes et al. 2016a; Liu and Locasale 2017; Worley and Powers 2013). Metabolomics techniques are applied to investigate the response to pathophysiological stimuli or genetic modifications (Lindon et al. 2000) such as cancer (Dang et al. 2009), diseases (Wild et al. 2019), or intoxications (D'Elia et al. 2019). Metabolomics has lately also been used to study DOA or NPS, either with a focus on the drug metabolism (Manier et al. 2019a; Mortele et al. 2018; Vervliet et al. 2019a) or the wider changes of the metabolome in abusers (Boxler et al. 2018). The huge potential of these techniques for the identification of biomarkers of DOA or NPS abuse can be found in the enhanced number of metabolites that might be detected and the identification of compounds that at first do not seem to be related to the drug (Manier et al. 2019a; Vervliet et al. 2019b). However, the toxicometabolomics, which means applying metabolomics to toxicology (Manier and Meyer 2020; Milburn et al. 2013), of DOA/NPS in HepaRG cells by means of metabolomics techniques has not been investigated yet. As such lines offer a comparable standardized biological environment, a high similarity to the actual physiology of the liver, and metabolites comparable to that of the microsomal fractions (Richter et al. 2017a), this approach appears to be promising.

The aims of study were therefore to investigate the toxicometabolomics of the two NPS α-pyrrolidinobutiophenone (1-phenyl-2-(pyrrolidin-1-yl)butan-1-one, α-PBP) and α-pyrrolidinoheptaphenone (1-phenyl-2-(pyrrolidin-1-yl)heptan-1-one, α-PEP, PV8) in HepaRG incubations. Pyrrolidinophenone-derived NPS are synthetic derivatives of the natural alkaloid cathinone. The primary amine moiety within the cathinone molecule is replaced by a pyrrolidine ring and the α-carbon is substituted with alkyl chains of different lengths. Consumption and seizure of these stimulants has been reported in Europe as well as Japan (EMCDDA 2015; Kudo et al. 2015; Odoardi et al. 2016; Swortwood et al. 2016). The general metabolism of the two NPS in, e.g., pHLM has so far been investigated (Manier et al. 2018, 2019a; Matsuta et al. 2015; Swortwood et al. 2016). The results shall be compared with those studies.

## Materials and methods

### Chemicals and reagents

α-PBP and α-PEP were provided by the Bavarian State Criminal Police Office (Munich, Germany). Water was purified with a Millipore filtration unit (which purifies water to a resistance of 18.2 Ω × cm). Cryopreserved, differentiated HepaRG cells, 96-well plates coated with type I collagen, fetal bovine serum, Williams’ E Medium, GlutaMAX, supplement HPRG620, and supplement HPRG670 were from Life Invitrogen (Darmstadt, Germany). Penicillin and streptomycin were purchased from Sigma-Aldrich (Taufkirchen, Germany), tryptophan-d_5_ was purchased from Alsachim (Illkirch Graffenstaden, France), acetonitrile (LC–MS grade), ammonium formate (analytical grade), formic acid (LC–MS grade), methanol (LC–MS grade), and all other chemicals and reagents (analytical grade) were from VWR (Darmstadt, Germany). Reaction tubes and pipette tips were obtained from Sarstedt (Nümbrecht, Germany).

### Incubations using HepaRG cells

Incubations using HepaRG cells were performed according to a previous publication (Richter et al. 2019). For each experiment, cells were maintained in an incubator (Binder, Tuttlingen, Germany) at 37 °C with 95% air humidity and 5% CO_2_ atmosphere. Cell handling was done under sterile conditions using a laminar flow bench class II (Thermo Scientific Schwerte, Germany). All given concentrations are final concentrations. Cryopreserved, differentiated HepaRG cells were cultivated according to the manufacturer’s instructions and seeded in a density of 80,000 cells/well in collagen-coated 96-well plates using Williams’ E medium supplemented with 1% GlutaMAX, HPRG670, 100 U/mL penicillin, 100 µg/mL streptomycin, and 0.5% DMSO on day 1. On day 2, this medium was replaced by the above described medium containing HPRG620 instead of ADD670 and subsequently renewed on day 5. On day 8, α-PBP and α-PEP were freshly dissolved in DMSO and subsequently diluted using the medium to the required concentration. The cell medium was finally renewed and additionally contained either 0 (group Blank), 12.5 (group Low) or 25 µM (group High) α-PBP or α-PEP, respectively. Each concentration was used in nine different wells. Medium volume was always 100 µL. After 24 h of exposure, the medium was transferred into a second 96-well plate that was not collagen coated. The cells were washed twice using 100 µL of phosphate-buffered saline (PBS buffer). Both plates were shock frosted using liquid nitrogen and subsequently stored at − 80 °C until analysis.

### Metabolomics sample preparation

After 3 days at − 80 °C, the cells were extracted (extract 1) on dry ice using a modified extraction method and recommendations published previously (Barnes et al. 2016b; Van den Eede et al. 2015). For this purpose, 100 µL of a precooled (− 80 °C) extraction mixture containing methanol and purified water (8:2, v/v) and 5 µM tryptophan-d_5_ as internal standard was added to the cells. The mixture was incubated for 15 min on dry ice until the content of each well was transferred into a reaction tube. This procedure was repeated once and both aliquots of each well were combined. All samples were shaken for 2 min at 2000 rpm and subsequently centrifuged for 30 min at 15,000 rpm and 2 °C. 150 µL of the supernatant was transferred into a new reaction tube and evaporated to dryness using a vacuum centrifuge (Concentrator plus, Eppendorf, Hamburg) at room temperature and program “V-aq” for roughly 1 h. The residue was reconstituted in 50 µL of a mixture containing methanol and acetonitrile (3:7, v/v) by shaking for 5 min at 15,000 rpm and 22 °C. Ten µL of each sample of both drugs of abuse were pooled to obtain one quality control sample (group QC) for every cell experiment.

For cell media extraction (extract 2), 70 µL of each of the cell media samples was transferred into a reaction tube and mixed with 210 µL precooled methanol (− 80 °C) containing 5 µM tryptophan-d_5_ by shaking for 2 min at 2000 rpm. 200 µL of each supernatant was transferred into a new reaction tube and evaporated to dryness under the above described conditions for roughly 2.5 h. Again, the residues were reconstituted in 50 µL of a mixture containing methanol and acetonitrile (3:7, v/v) by shaking for 5 min at 15,000 rpm and 22 °C. Ten µL of each sample of both drugs was pooled to obtain quality control samples (group QC) for every medium experiment.

Every obtained sample was transferred into an amber glass vial and 1 µL was injected onto the HPLC–HRMS/MS as described in the corresponding paragraph.

### Incubation and sample preparation for investigation of imine formation

To confirm the formation of imines by α-PBP and α-PEP when being incubated with glycine, several conditions were chosen based on previous studies (Welter et al. 2013). Glycine concentration was based on its concentration in the Williams’ E medium. α-PBP and α-PEP were freshly dissolved and subsequently diluted using either purified water, Williams’ E medium, or Tris buffer to obtain the required concentrations prior to the experiment. The same was done for glycine, except for the dilution in Williams’ E medium. Incubations were conducted at 37 °C for 24 h using 25 µM α-PBP or α-PEP in either Tris buffer containing 0 or 666 µM glycine, Williams’ E medium, or purified water containing 0 or 666 µM glycine. Additionally, all incubations were performed without α-PBP and α-PEP containing 0 or 666 µM glycine. Incubation volume was 50 µL, respectively. Each experiment was performed in triplicate. After incubation, 10 µL of 25% formic acid in purified water was added to the samples, as well as 40 µL of Eluent F as described in the following paragraph to obtain pH 3 in all samples. A 10-µL aliquot was subsequently injected onto the HPLC–HRMS/MS.

### LC–HRMS/MS apparatus

The analysis was performed using a Thermo Fisher Scientific (TF, Dreieich, Germany) Dionex UltiMate 3000 RS pump consisting of a degasser, a quaternary pump, and an UltiMate Autosampler, coupled to a TF Q-Exactive Plus system equipped with a heated electrospray ionization HESI-II source. Mass calibration was done prior to analysis according to the manufacturer’s recommendations using external mass calibration. Additionally before each experiment, the spray shield and capillary were cleaned. The performance of the column and the mass spectrometer was tested using a mixture as described by Maurer et al. (2016, 2018) prior to every experiment. The conditions were set according to published procedures (Helfer et al. 2015; Wagmann et al. 2017). Gradient reversed-phase elution was performed on a TF Accucore PhenylHexyl column (100 mm × 2.1 mm, 2.6 µm, TF, Dreieich, Germany) or on a hydrophilic interaction liquid chromatography (HILIC) Nucleodur column (125 × 3 mm, 3 μm, Macherey–Nagel, Düren, Germany) for normal-phase chromatography. The mobile phases for gradient elution using the PhenylHexyl column consisted of 2 mM aqueous ammonium formate containing acetonitrile (1%, v/v) and formic acid (0.1%, v/v, pH 3, eluent A), as well as 2 mM ammonium formate in acetonitrile and methanol (1:1, v/v), containing water (1%, v/v) and formic acid (0.1%, v/v, eluent B). The flow rate was set from 1–10 min to 500 µL/min and from 10–13.5 min to 800 µL/min using the following gradient: 0–1.0 min hold 99% A, 1–10 min to 1% A, 10–11.5 min hold 1% A, 11.5–13.5 min hold 99% A. Normal-phase chromatography was performed using aqueous ammonium acetate solution (200 mM, eluent C) and acetonitrile containing formic acid (0.1%, v/v, eluent D). For chromatography of imines, eluent C was replaced by aqueous ammonium formate solution (20 mM, eluent E) adjusted to pH 3 using formic acid and eluent D was replaced with a mixture of 200 mM aqueous ammonium formate and acetonitrile (1:10, v/v, eluent F). These experiments were performed on the HILIC column using the positive ionization mode. The flow rate was set to 500 µL/min using the following gradient: 0–1 min 2% C, 1–5 min to 20% C, 5–8.5 min to 60% C, 8.5–10 min hold 60% C, 10–12 min hold 2% C. For preparation and cleaning of the injection system, a mixture containing isopropanol and water (90:10, v/v) was used. The following settings were used: wash volume, 100 µL; wash speed, 4000 nL/s; loop wash factor, 2. Every analysis was performed at 40 °C column temperature, maintained by a Dionex UltiMate 3000 RS analytical column heater. The injection volume for metabolomics analyses was 1 µL and for those analyses investigating the formation of imines 10 µL. The HESI-II source conditions for every experiment were as follows: ionization mode, positive or negative; sheath gas, 60 AU; auxiliary gas, 10 AU; sweep gas, 3 AU; spray voltage, 3.50 kV in positive mode and -4.0 kV in negative mode; heater temperature, 320 °C; ion transfer capillary temperature, 320 °C; and S-lens RF level, 50.0. Mass spectrometry for untargeted metabolomics (UM) was performed according to a previously optimized workflow using full scan (FS) only (Manier et al. 2019b). The settings for FS data acquisition were as follows: resolution, 140,000 fwhm; microscans, 1; automatic gain control (AGC) target, 5 × 10^5^; maximum injection time, 200 ms; scan range, *m/z* 50–750; polarity, negative or positive; spectrum data type, centroid.

The settings for parallel reaction monitoring (PRM) data acquisition were as follows: resolution, 35,000 fwhm; microscans, 1; AGC target, 5 $\times$ 10^5^; maximum injection time, 200 ms; isolation window, 1.0 m*/z*; collision energy (CE), 10, 20, or 40 eV; spectrum data type, centroid. The inclusion list contained the monoisotopic masses of all significant features and a time window of their retention time $\pm$ 30 s. Analysis was performed using a randomized sequence order with five injections of pure methanol (reversed-phase chromatography) or eluent D (normal-phase chromatography) samples at the beginning of the sequence for apparatus equilibration, followed by five injections of the pooled QC sample. Additionally, one QC injection was performed every five samples to monitor batch effects as described by Wehrens et al. (2016).

Analysis concerning additional incubations to confirm the formation of imines was performed using full scan (FS) data and subsequent data-dependent acquisition (DDA). The settings for FS data acquisition were as follows: resolution, 35,000; microscans, 1; automatic gain control (AGC) target, 1e6; maximum injection time, 120 ms; and scan range, *m/z* 50–50. The settings for the DDA mode were as follows: dynamic exclusion, 0.1 s; resolution, 17,500; microscans, 1; loop count, 5; AGC target, 2e4; maximum injection time, 250 ms; isolation window, *m/z* 1.0; high collision dissociation (HCD) with stepped normalized collision energy (NCE), 17.5, 35, and 52.5%; spectrum data type, profile; and underfill ratio, 1%. Additionally, an inclusion list containing *m/z* values of the suspected adducts was used, although MS^2^ experiments were not limited to these (if idle, pick others). TF Xcalibur software version 3.0.63 was used for all data handling.

### Data processing

Thermo Fisher LC-HRMS/MS RAW files were converted to mzXML format using Proteo Wizard (Adusumilli and Mallick 2017). Peak picking was performed using XCMS in an R environment (Smith et al. 2006; Team R Core Team); annotation of isotopes, adducts, and artifacts was performed using the R package CAMERA (Kuhl et al. 2012). Optimization of XCMS parameters was in accordance with a previously optimized strategy (Manier et al. 2019b). Peak picking and alignment parameters are summarized in Table S1. According to Wehrens et al. (2016), feature abundances with a value of zero were replaced by the lowest measured abundance as a surrogate LOD and subsequently log10 transformed. A batch correction was performed for those features that were detected in every QC sample. The corresponding feature abundance was corrected using a linear model to extrapolate its abundance drift between QC samples (Wehrens et al. 2016). Additionally, all feature areas were normalized to the area of tryptophan-d_5_, to remove unwanted variation, e.g., from pipetting − 80 °C extraction mixtures. Subsequently, a corresponding p value was calculated using a one-way analysis of variance (ANOVA) and insignificant features were removed from the data set. The data set was filtered keeping merely those features with a *p*-value of < 0.001. Patterns in the data set were subsequently investigated using principal component discriminant function analysis (PC-DFA) and hierarchical clustering. For principal component analysis, the features were centered. The subsequent discriminant analysis was performed using those principal components that fulfilled Kaisers's criterion, but at least two. The quality of the model was assessed using its accuracy of predicting group membership, as well as Cohen’s *κ* after Monte Carlo cross-validation. Cohen’s *κ* was interpreted as proposed by Landis and Koch (Landis and Koch 1977). Hierarchical clustering was performed after row scaling, using Euclidean distances. The names of the features were adopted from XCMS using “M” followed by the rounded mass and “T” followed by the retention time in seconds (e.g., “M218T222” as given in Table S2 for protonated α-PBP at *m/z* 218.1538 and a retention time of 222 s by reversed-phase chromatography).

### Identification of significant features

MS^2^ spectra were recorded using the above-mentioned PRM method to allow identification of significant features. Individual spectra were exported after subtracting the baseline left and right of the peak. After conversion to mzXML format using Proteo Wizard, spectra were imported to NIST MSSEARCH version 2.3. A library search for identification was conducted using the following settings: spectrum search type, identity (MS/MS); precursor ion *m/z*, in spectrum; spectrum search options, none; presearch, off; other options, none. MS/MS search was conducted using the following settings: precursor tolerance, ± 5 ppm; product ion tolerance, ± 10 ppm; ignoring peaks around precursor, ± *m/z* 1. The search was conducted by using the following libraries: NIST 14 (nist_msms and nist_msms2 sublibraries) and Wiley METLIN Mass Spectral Database. Metabolites of the investigated NPS were identified by comparing and interpreting their spectra to those of the parent compounds.

## Results

Data files in mzXML format and the R script files can be found at www.github.com/saskema/hepargmetabolomics. The MS^2^ spectra of significant features, available as indicated in Table S2–S5 in the supplementary data, can be found as well on the above-mentioned repository in mzXML format.

### Untargeted metabolomics for significant feature detection

**Fig. 1:**
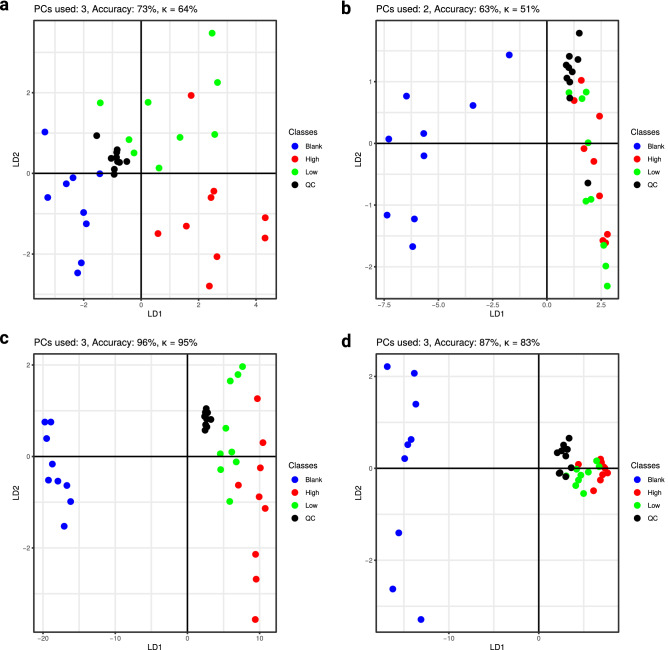
Scores of PC-DFA for α-PBP with the number of principal components used for discriminant function analysis, as well as prediction accuracy and Cohen’s *κ*. **a** Extract 1 using phenylhexyl column and positive mode; **b** Extract 1 using HILIC column and positive mode, **c** Extract 2 using phenylhexyl column and positive mode, **d** Extract 2 using HILIC and positive mode, *PC* = principal component,* LD* = linear discriminant

**Fig. 2:**
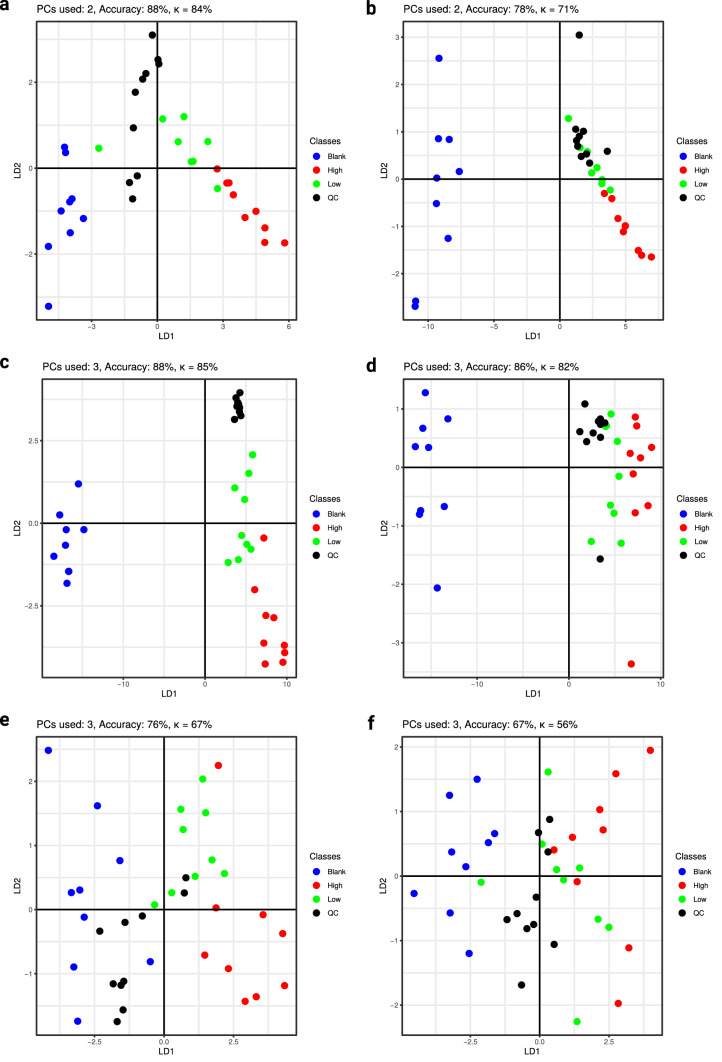
Scores of PC-DFA for α-PEP with the number of principal components used for discriminant function analysis, as well as prediction accuracy and Cohen’s *κ*. **a** Extract 1 using phenylhexyl column and positive mode; **b** Extract 1 using HILIC column and positive mode, **c** Extract 2 using phenylhexyl column and positive mode, **d** Extract 2 using HILIC and negative mode, **e** Extract 2 using phenylhexyl column and negative mode, **f** Extract 2 using HILIC and negative mode. *PC* = principal component, *LD* = linear discriminant

The results of the ANOVA can be found in Figures S1 and S2, scores of the PC-DFA are displayed in Figs. 1 and 2, and the corresponding loadings in Figures S3 and S4. The results of the hierarchical clustering are displayed in heatmaps in Figures S5 and S6. Due to a technical error of the autosampler, one analysis of α-PEP (+ HepaRG) had to be excluded from statistical evaluation.

Only few features were found significant after analyses of extract 1 (Figure S1 a-d and Figure S2 a-d). For incubations with α-PBP, four significant features were found after reversed-phase chromatography and positive ionization and two significant features after normal-phase chromatography and positive ionization, as well as one after analysis using normal-phase chromatography and negative ionization (Figures S1 a-d). Analyses using reversed-phase chromatography and negative ionization did not lead to any significant feature. Concerning extract 1 after α-PEP incubation, any analysis using positive ionization led to two significant features, while negative ionization did not lead to any significant feature. Inspection of the extracted ion chromatograms did not imply a high variance within incubation groups, but a lack of variance between them, which led to negative ANOVA results. Analyses of extract 2 revealed far more significant features. For α-PBP, analyses using positive ionization and reversed-phase chromatography resulted in seven significant features and using normal-phase chromatography six. Analyses using negative ionization did not expose any significant changes. Analyses of cell media using positive ionization after incubations with α-PEP led to the discovery of 14 significant features after either using reversed- or normal-phase chromatography. Negative ionization analyses led to four significant features after reversed-phase chromatography column and three after using normal-phase chromatography. After filtering the data set and evaluation using PC-DFA, these features showed a consistent clustering of incubation groups in each experiment (Figs. 1, 2). Throughout all score plots, those incubation groups containing the corresponding NPS are clearly separated from the group “Blank”. Since pooled QC samples were obtained from mixing all three incubation groups containing equidistant concentrations of the NPS, they contained a concentration approximately corresponding to that of the incubation group “Low”. This circumstance led to overlapping of both groups in most of the displayed score plots.

Concerning the analysis of extract 1, the accuracy of the PC-DFA model was 73% for incubations with α-PBP after analysis using reversed-phase chromatography and 63% after using normal-phase chromatography. Corresponding κ values were 64% and 51%, respectively. Concerning α-PEP, κ values obtained after cross-validation imply good discriminant properties for features found in cell samples with 84% after using reversed-phase chromatography and 71% after using normal-phase chromatography.

The quality of the PC-DFA models was best after analysis of extract 2 in positive mode, where at least an accuracy of 86% and Cohen’s *κ* of 82% (category “almost perfect” according to Landis and Koch) were achieved. Models of cell media experiments (extract 2) of α-PEP that were analyzed in negative mode and reversed-phase chromatography led to a *κ* value of 67%, which might be categorized as “substantial” according to Landis and Koch, while corresponding experiments using normal-phase chromatography led to a rather low *κ* value of 56%. The results of hierarchical clustering corresponded to those found after PC-DFA. Almost all samples from group “Blank” were clustered and separated from all other samples containing one of the investigated NPS. The Euclidian distance of “Blank” group to other samples and the distances between other incubation groups were higher in experiments investigating extract 2, than in extract 1. Furthermore, groups of extract 2 analyzed in negative ionization mode (Figure S6 e and f) were less clearly separated than in positive mode. Also, the z-scores of the peak intensities indicated discrimination of the groups corresponding to hierarchical clustering and PC-DFA scores.

### Identification of significant features

The results of the identification of significant features are summarized in Tables S2-5 in the supplementary data. The given level of identification was in accordance with Sumner et al. (2007). Isotopes that were putatively identified by CAMERA were not further identified. No MS^2^ spectra could be recorded for several low abundant features.

### Extract 1

Mainly, the parent compounds and their ^13^C-isotopes were found after α-PBP and α-PEP incubations. Several additional features showed significant changes in α-PBP incubations, but only the ^13^C-isotope of *N*-methylnicotinamide and the ammonium adduct of decaethylene glycol could successfully be identified. Decaethylene glycol was identified by a partial match with hexaethylene glycol. Since the structure of polyethylene glycols is highly repetitive, the polymer length was able to be deduced from the protonated parent mass of hexaethylene glycol at *m/z* 282.1678 (C_12_H_26_O_7_). The structure of the corresponding feature M476T250 is thus deducible by combination of components of a polyethylene glycol polymer and ammonium as 10 × *m/z* 44.0261 (C_2_H_4_O, repeating unit) + *m/z* 18.0105 (H_2_O, end group) + *m/z* 17.0263 (NH_4_^+^, adduct ion) = *m/z* 476.3071 (C_20_H_46_NO_11_).

### Extract 2

**Fig. 3:**
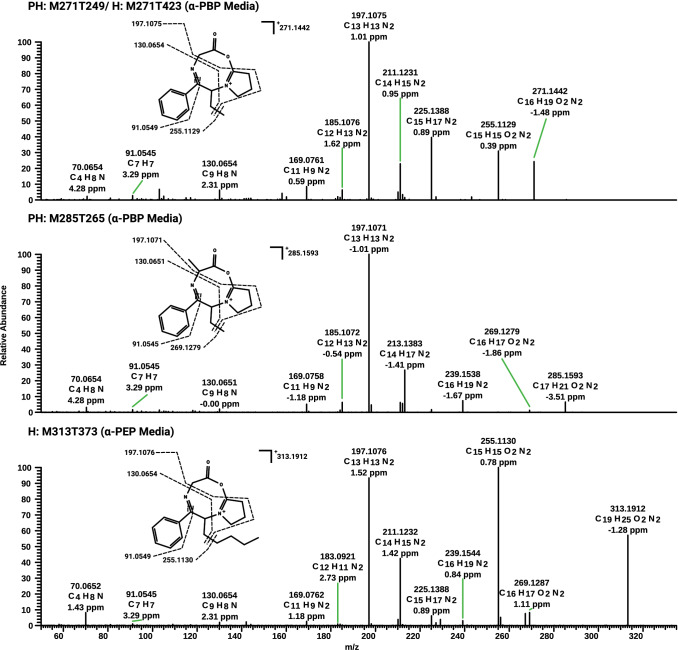
LC–HRMS/MS spectra of amino acid adducts and their predominant fragmentation patterns after incubations with α-PBP or α-PEP. Fragments with accurate mass, calculated elemental formula, and mass error value in parts per million (ppm). The order corresponds to their masses

A total of 11 out of 14 features found after α-PBP incubation were related to the parent compound. Four features were ^13^C-isotopes of the parent compound, while two features were related to one metabolite, found after analysis using each of the chromatographic methods. Additionally, two features were identified to be most likely amino acid adducts of α-PBP with glycine and alanine, respectively (Table S3). Their MS^2^ spectra and the proposed structures are displayed in Fig. 3. Since this feature was only found in incubations that contained α-PBP and absent in those of group “Blank”, a formation of this feature from the NPS itself seemed very likely. The fragmentation patterns of these two features indicated a shared basic structure, since both spectra contained the same main fragment ions concerning lower masses, starting with the fragment ion at *m/z* 197.1073 (C_13_H_13_N_2_). It is also worth noticing that both protonated parent compounds at *m/z* 271.1446 (C_16_H_19_N_2_O_2_) for α-PBP glycine adduct and at *m/z* 285.1603 (C_17_H_21_N_2_O_2_) for α-PBP alanine adduct merely differ by one methyl group in mass and deduced sum formula. Additionally, their absolute loadings implied that the contribution of these features (M271T249 and M285T265 in Figure S3 c, M271T423 in Figure S3 d) to group separation was as high as that of a metabolite (M220T217 in Figure S3 c and M220T319 in Figure S3 d). The amount of metabolites and adducts of α-PBP that were identified in cell media samples explained the higher discriminating properties of these features, as indicated by Cohen’s κ which was 95% for those found after analysis using reversed-phase chromatography (Fig. 1c) and 83% for those found after analysis using normal-phase chromatography, compared to 64% and 51% for extract 1, respectively.

Analysis of samples after α-PEP incubation 2 allowed identification of the parent compound, five metabolites, as well as one glycine adduct and three more compounds not related to α-PEP. Cholesterol sulfate and 25-hydroxycholesterol were two of the identified compounds, indicating an increased cholesterol metabolism. The proposed structure and MS^2^ spectra of α-PEP glycine adduct are also displayed in Fig. 3. Compared to the spectra of the other adducts, the spectrum again contained the same main fragment ions with regard to lower masses, starting with the fragment ion at *m/z* 197.1073 (C_13_H_13_N_2_), indicating a shared basic structure of all three compounds. Additionally, if compared to the spectrum of the α-PBP glycine adduct, both spectra share the same main fragment ions starting with the second main fragment ion at *m/z* 255.1128 (C_15_H_15_N_2_O_2_). Considering these obvious similarities and the fact that both glycine adducts merely differ in their protonated parent mass by the difference as α-PBP and α-PEP do, it is very likely that these features indicate actual amino acid adducts. The feature M51T284 in Table S5 could not further be investigated due to the mass cutoff of *m/z* 50 of the used mass spectrometer. Nevertheless, concerning its mass and retention time, it is likely that this feature is an artifact of α-PEP and was thus annotated in Table S5. The feature M465T121 did not show any fragmentation after using 10, 20, or 40 eV. The only compound in the used mass spectra libraries for identification that had the same monoisotopic mass and did not show any fragmentation at the given collision energies was cholesterol sulfate. Since higher collision energies than 40 eV lead to not detecting any signal, it was not possible to further confirm its identity. The feature M338T87 in Table S5 was identified as oleamide.

### Incubations for further investigating the formation of imines

To confirm the formation of the adducts of the investigated NPS with glycine, further incubations were performed to confirm the findings described above under simplified conditions. A lower pH in the eluent was used to be able to detect glycine. The analysis of the obtained samples revealed that both adducts were formed in each incubation containing one of the investigated compounds with glycine and Tris buffer or cell medium. In incubations with α-PBP and cell media, the alanine adduct was found as well. None of the adducts were found after incubations using purified water instead of buffer solution. Additionally, no adduct of α-PEP and alanine was found in incubations with cell medium only.

## Discussion

This study revealed different discriminant properties of extract 1 and 2 after PC-DFA. Since it is a multi-class study, rather than merely investigating the classification in a blank or treatment group, the evaluation of Cohen’s κ is more appropriate, due to the fact that it resembles the accuracy of the model normalized to the accuracy one would obtain by chance (Landis and Koch 1977). Nevertheless, the prediction accuracy of the models was also given for completeness.

κ values for analyses extract 1 would be classified as “moderate” according to Landis and Koch (Landis and Koch 1977), indicating that they have to be interpreted with care, while those of extract 2 can be generally classified as “almost perfect”.

Those identified compounds that are not related to the investigated NPS, give a hint to their effect on physiology of the used liver cells. *N*-Methylnicotinamide is linked to liver cirrhosis and might indicate a liver toxic effect of α-PBP (Pumpo et al. 2001). The reason why merely its ^13^C-isotope and not *N*-nicotinamide itself was found significant after ANOVA is likely due to a high within-group variance. The interpretation of the role of the two found cholesterol metabolites is not possible without speculating, since both metabolites are not directly connected to each other. The occurrence of polyethylene glycols is rather peculiar. Since they are not natural endogenous compounds of cells, it is very likely that this feature is the result of contamination by a lubricant. During experiments, no lubricant was used and it appears to be likely that this contamination happened during analysis by the used device. The origin of oleamide on the other side is not clearly determinable. Although this substance is a naturally occurring endogenous compound, McDonald et al. (2008) earlier described that several plasticware contains it as a slip agent. They also demonstrated that oleamide is detectable in the water these plasticware were rinsed with. Given the fact that the plasticware used in this study were among those tested by McDonald et al. and the ion chromatograms of oleamide showed high variability within each incubation group, it is very likely that the detected oleamide is not of endogenous origin, but a leached slip agent.

**Fig. 4:**
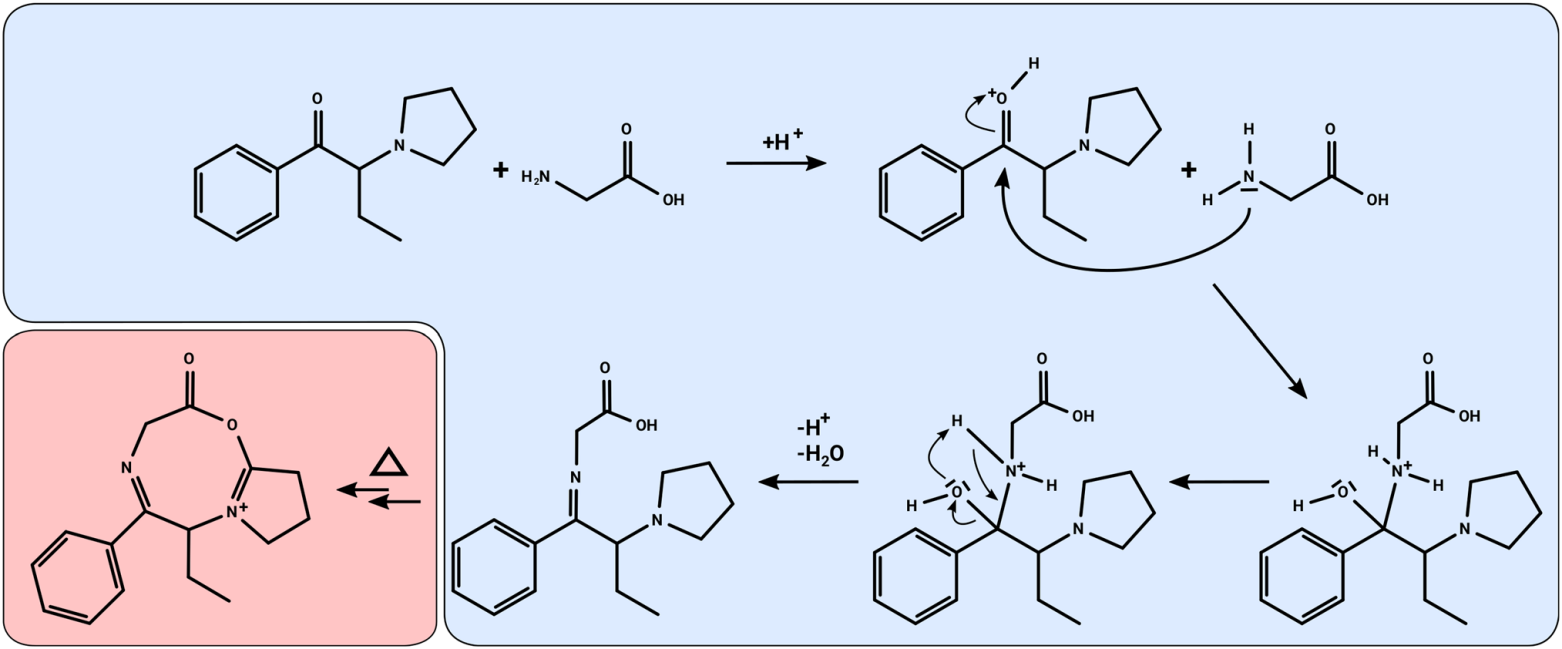
Proposed mechanism of the formation of amino acid adducts exemplified for α-PBP and glycine. Blue colored part is presumably occurring during incubation. The red part presumably occurs in heated electrospray ionization source or in transfer capillary of the mass spectrometer (color figure online)

It is most likely that the investigated NPS formed imines with amino acids during incubations. This formation might be achieved after nucleophilic attack of the amine moiety of the amino acid at the cathinone carbonyl group. Since incubations using only Tris buffer did not contain any enzymes, the ring is likely to be formed during analysis in the ion source. It is notable that not every incubation using only NPS and amino acid lead to the detection of these adducts. The absence of adducts in those incubations using purified water might indicate a pH dependency of the formation, while the absence of the alanine adduct with α-PEP might be due to the fact that it was formed with too low concentrations to be detectable in this assay. A summary of the proposed mechanism is displayed in Fig. 4. The formation of such an adduct also in vivo is likely, since the used concentrations of glycine and alanine are similar to those concentrations that can be found in human blood (Psychogios et al. 2011).

The metabolism of α-PBP and α-PEP has been thoroughly investigated in previous publications using either pHLM (Manier et al. 2018, 2019a), primary human hepatocytes (Swortwood et al. 2016), or authentic human urine (Matsuta et al. 2015). α-PBP was described to be transformed to five metabolites, one after reduction of the cathinone carbonyl group resulting in a dihydro metabolite and several others after oxidation of the pyrrolidine ring, resulting in a mono and dihydroxylated metabolite, as well as an oxo and a ring-opened hydroxy metabolite. HepaRG did only form the dihydro metabolite, one of the main metabolites of α-PBP (Manier et al. 2018). Oxidation of the pyrrolidine ring was not observed, although, for example, the metabolite found after lactam formation was also described as the second main metabolite. This is most probably due to fact that the HepaRG cell line was derived from a donor known to be a CYP2D6 poor metabolizer (Guillouzo et al. 2007). Investigations of the CYP isoforms involved in the metabolism of α-PBP revealed that lactam formation of α-PBP was only catalyzed by CYP2D6 (Manier et al. 2018). Additionally, pyrrolidine ring hydroxylation was catalyzed by CYP2B6, CYP2C19, and CYP2D6, of which CYP2D6 is expressed to a reduced extent due to the cells being derived from a poor metabolizing patient and CYP2B6 has a low contribution to the hepatic total net clearance of α-PBP. It is very likely that the number of metabolites formed after oxidation of the pyrrolidine ring was too low to be detected. This assumption is supported by the generally low amount of CYP2C19 expressed in human liver (Achour et al. 2014). The formation of amino acid adducts was not mentioned in any of the previously published studies, although the “α-PBP impurity (dehydro-) artifact (imido-)” described by Manier et al. might represent another in-source artifact of an adduct formed after the mechanism proposed in this study.

Concerning the metabolism of α-PEP, only five of the nine metabolites described by Manier et al. (2019a) were found after incubation of HepaRG cells, although its metabolism is catalyzed by a wide variety of CYP enzymes such as CYP1A2, CYP2B6, CYP2C9, CYP2C19, CYP2D6, and CYP3A4. Lactam formation, dihydroxylation, hydroxylation of the side chain, hydroxylation after opening of the pyrrolidine ring, and reduction of the cathinone carbonyl group were observed. Single pyrrolidine ring hydroxylation, dealkylation, and several combinations were not detected. Metabolites after *N-*oxidation, ring opening and formation of a carboxylic acid, and dehydrogenation as described by Swortwood et al. (2016) were also not detected. Nevertheless, alkyl hydroxylation, lactam formation, and reduction of the cathinone carbonyl group were described as the main metabolic steps of α-PEP and were detected after incubation with pHS9/pHLM (Manier et al. 2018). Again, adduct formation of α-PEP with glycine was not mentioned by any of the previously published studies.

Since most metabolites were mainly found in extract 2 (cell media) and HepaRG cells were described to express functional transporter proteins, it is likely that they were actively transported (Guillouzo et al. 2007). Although metabolomics studies using pHLM led to higher amounts of metabolites of the investigated drugs of abuse, incubations using HepaRG cells led to more significant compound changes that were not directly linked to the parent compounds. This might give a wider insight into the physiological impact on the liver cell metabolome. Depending on the aims of the study, it seems more appropriate to use pHLM for the investigation of metabolic pathways. If the aims of the study are to investigate the direct effect of compounds on the physiology of the cells such as hepatotoxicity, it seems more appropriate to use HepaRG cells.

## Conclusions

Toxicometabolomics of the two NPS α-PBP and α-PEP after HepaRG cell line incubation were evaluated by analyzing the cells and the cell medium separately. The cross-validation results of the cell media lead to the conclusion that the features found in these experiments had better discriminant properties and thus are more suitable as biomarkers for the intake of the NPS investigated by this study. Significant features found in the cell extracts were mainly the parent compounds and their corresponding ^13^C-isotopes. Significant features found in the cell media led to a considerably higher amount of significant changes in the metabolome that were identified as metabolites of both synthetic cathinones, as well as amino acid adducts but also remained unknown several features due the lack of reference spectra. They might give a wider insight into the physiological changes of the cells, since they were not able to be linked to the investigated cathinones. So far, only cholesterol sulfate and 25-hydroxycholesterol could be identified to have significantly changed in concentrations after incubation of α-PEP, as well as *N*-methylnicotinamide in cell samples after incubation with α-PBP. These increased levels of *N*-methylnicotinamide may indicate a potential liver toxic effect of α-PBP. The investigation of the metabolism of the two synthetic cathinones led to fewer metabolites compared to investigations using pHLM or primary human hepatocytes, partially owing to the fact that HepaRG cells are derived from a CYP2D6-poor metabolizing patient. The number of metabolites found in α-PBP and α-PEP HepaRG incubated using metabolomics techniques may lead to two conclusions. HepaRG cells are not appropriate to investigate the metabolism of compounds mainly metabolized by CYP2D6. Metabolomics techniques are not able to extract all formed metabolites, particularly if they are very low abundant. The second conclusion could at least be excluded in the present study, as manual inspection of the data also did not allow identification of the missed metabolites.

The results of the conducted experiments, especially those of the incubations of the NPS with amino acids, revealed that both NPS formed the highly discriminating but unexpected imines with the corresponding amino acid during incubations. Their detection underlined the potential of toxicometabolomics studies.

## Electronic supplementary material

Below is the link to the electronic supplementary material.

Supplementary file1 (PDF 1466 kb)
