# Supplementary material for: Toxicometabolomics of the new psychoactive substances α-PBP and α-PEP studied in HepaRG cell incubates by means of untargeted metabolomics revealed unexpected amino acid adducts
Source: Arch Toxicol. 2020 Apr 20;94(6):2047–59. doi: 10.1007/s00204-020-02742-1 (PMC7303098; doi:10.1007/s00204-020-02742-1)
Supplement: Supplementary file 1 — Supplementary file1 (PDF 1466 kb) [file 204_2020_2742_MOESM1_ESM.pdf]

Toxicometabolomics of the new psychoactive substances α-PBP and α-PEP studied in HepaRG cell incubates by means of untargeted metabolomics revealed unexpected amino acid adducts

Electronic Supplementary Material

Sascha K. Manier, Lea Wagmann, Veit Flockerzi, Markus R. Meyer

Table S1 Peak picking and alignment parameters used for preprocessing. PH = PhenylHexyl, HI = HILIC, pos = positive, neg = negative, ppm = allowed ppm deviation of mass traces for peak picking, snthresh = signal to noise threshold, mzdiff = minimum difference in *m/z* for two peaks to be considered as separate, prefilter 1 = minimum of scan points, prefilter 2 = minimum abundance, bw = bandwidth for grouping of peaks across separate chromatograms.

Experiment	Column	Polarity	peakwidth, min, s	peakwidth, max, s	ppm	snthresh	mzdiff	prefilter 1	prefilter 2	bw
Extract 1	PH	pos	7.8	17	1.1	33	0.002	6	52000	1.2
Extract 1	PH	neg	9.5	94	1.2	71	-0.1	4	1000	0.6
Extract 1	HI	pos	8.9	33	1.1	5	0.01	9	71000	0.7
Extract 1	HI	neg	7.8	75	1	47	0.022	14	8000	0.7
Extract 2	PH	pos	7.8	27	1.2	20	0.001	7	74000	0.9
Extract 2	PH	neg	7.8	100	1.5	49	0.094	8	20000	0.7
Extract 2	HI	pos	8.9	100	1.1	44	0.01	15	17000	0.7
Extract 2	HI	neg	7.8	82	1.8	34	0.052	1	1000	0.7

Table S2 Significant features found in extract 1 after incubation with α-PBP. Features ordered by column type, ionization mode, mass per charge (*m/z*) and retention time. LOI = level of identification, RT = retention time,

eV = electron volt, PH = PhenylHexyl, HI = HILIC, pos = positive, neg = negative, x = available, / = not analyzed,

n.d. = not detectable.

Column type	Ionization mode	Feature	Identity	LOI	*m/z*	RT, s	Available MS^2 Spectrum, eV		
							10	20	40
PH	pos	M138T32	*N*-Methylnicotinamide ^{13}C-Isotope	3	138.0743	32	/	/	/
PH	pos	M155T31	Unknown	4	154.8803	31	n.d.	n.d.	n.d.
PH	pos	M218T222	α-PBP	2	218.1538	222	x	x	x
PH	pos	M476T250	Decaethylenglycol $[M+NH_4]^+$	2	476.3065	250	x	x	x
HI	pos	M218T318	α-PBP	2	218.1543	318	x	x	x
HI	pos	M219T319	α-PBP ^{13}C-Isotope	3	219.1577	319	/	/	/
HI	neg	M196T377	Unknown	4	195.8102	377	x	x	x

Table S3 Significant features found in extract 2 after incubation with α-PBP. Features ordered by column type, ionization mode, mass per charge (*m/z*) and retention time. LOI = level of identification, RT = retention time, eV = electron volt, PH = PhenylHexyl, HI = HILIC, pos = positive, neg = negative, x = available, / = not analyzed, n.d. = not detectable.

Column type	Ionization mode	Feature	Identity	LOI	m/z	RT, s	Available MS^2 Spectrum, eV		
							10	20	40
PH	pos	M82T30	Unknown	4	82.02710	30	x	nd	nd
PH	pos	M218T217	α-PBP	2	218.15422	217	x	x	x
PH	pos	M219T217	α-PBP ^{13}C-Isotope	3	219.15748	217	/	/	/
PH	pos	M220T217	α-PBP $^{13}C_2$-Isotope	3	220.16075	217	/	/	/
PH	pos	M220T226	α-PBP-M (Dihydro-)	3	220.16977	226	x	x	x
PH	pos	M271T249	α-PBP Glycine Adduct	3	271.14422	249	x	x	x
PH	pos	M285T265	α-PBP Alanine Adduct	3	285.15984	265	x	x	x
HI	pos	M157T114	Unknown	4	157.0432	114	x	x	x
HI	pos	M218T319	α-PBP	2	218.1543	319	x	x	x
HI	pos	M219T319	α-PBP ^{13}C-Isotope	3	219.1576	319	/	/	/
HI	pos	M220T319	α-PBP $^{13}C_2$-Isotope	3	220.1609	319	/	/	/
HI	pos	M220T330	α-PBP-M (Dihydro-)	3	220.1699	330	x	x	x
HI	pos	M271T423	α-PBP Glycine Adduct	3	271.1444	423	x	x	x

Table S4 Significant features found in extract 1 after incubation with α-PEP. Features ordered by column type, ionization mode, mass per charge (*m/z*) and retention time. LOI = level of identification, RT = retention time, eV = electron volt, PH = PhenylHexyl, HI = HILIC, pos = positive, neg = negative, x = available, / = not analyzed, n.d. = not detectable.

Column type	Ionization mode	Feature	Identity	LOI	m/z	RT, s	Available MS^2 Spectrum, eV		
							10	20	40
PH	pos	M260T316	α-PEP	2	260.2007	316	x	x	x
PH	pos	M261T316	α-PEP ^{13}C-Isotope	3	261.2040	316	/	/	/
HI	pos	M260T316	α-PEP	2	260.2013	285	x	x	x
HI	pos	M261T316	α-PEP ^{13}C-Isotope	3	261.2046	285	/	/	/

Table S5 Significant features found in extract 2 after incubation with α-PEP. Features ordered by column type, ionization mode, mass per charge (*m/z*) and retention time. LOI = level of identification, RT = retention time, eV = electron volt, PH = PhenylHexyl, HI = HILIC, pos = positive, neg = negative, x = available, / = not analyzed, n.d. = not detectable.

Column type	Ionization mode	Feature	Identity	LOI	m/z	RT, s	Available MS^2 Spectrum, eV		
							10	20	40
PH	pos	M180T191	Unknown	4	180.0656	191	x	x	x
PH	pos	M260T311	α-PEP	2	260.2011	311	x	x	x
PH	pos	M261T311	α-PEP ^{13}C-Isotope	3	261.2044	311	/	/	/
PH	pos	M262T311	α-PEP $^{13}C_2$-Isotope	3	262.2078	311	/	/	/
PH	pos	M262T322	α-PEP-M (Dihydro-)	3	262.2167	322	x	x	x
PH	pos	M263T322	α-PEP-M (Dihydro-) ^{13}C-Isotope	3	263.2201	322	/	/	/
PH	pos	M274T429	α-PEP-M (Oxo-)	3	274.1803	429	x	x	x
PH	pos	M275T429	α-PEP-M (Oxo-) ^{13}C-Isotope	3	275.1836	429	/	/	/
PH	pos	M276T299	α-PEP-M (HO-) isomer 2	3	276.1960	299	x	x	x
PH	pos	M278T305	α-PEP-M (dihydro-HO-)	3	278.2117	305	x	x	x
PH	pos	M292T308	α-PEP-M (di-HO-)	3	292.1908	308	x	x	x
PH	pos	M293T308	α-PEP-M (di-HO-) ^{13}C-Isotope	3	293.1941	308	/	/	/
PH	pos	M299T329	α-PEP Glycine Adduct	3	299.2120	329	x	x	x
PH	pos	M367T647	25-Hydroxycholesterol [M+H]-H_2O	2	367.3363	647	x	x	x
HI	pos	M51T284	α-PEP Artifact	3	50.9809	284	n.d.	n.d.	n.d.
HI	pos	M260T285	α-PEP	2	260.2012	285	x	x	x
HI	pos	M261T285	α-PEP ^{13}C-Isotope	3	261.2046	285	/	/	/
HI	pos	M262T284	α-PEP $^{13}C_2$-Isotope	3	262.2079	284	/	/	/
HI	pos	M262T306	α-PEP-M (Dihydro-)	3	262.2168	306	x	x	x
HI	pos	M263T306	α-PEP-M (Dihydro-) ^{13}C-Isotope	3	263.2202	306	/	/	/
HI	pos	M274T85	α-PEP-M (Oxo-)	3	274.1805	85	x	x	x
HI	pos	M275T85	α-PEP-M (Oxo-) ^{13}C-Isotope	3	275.1837	85	/	/	/
HI	pos	M276T349	α-PEP-M (HO-) isomer 2	3	276.1961	349	x	x	x
HI	pos	M292T344	α-PEP-M (di-HO-)	3	292.1909	344	x	x	x
HI	pos	M293T344	α-PEP-M (di-HO-) ^{13}C-Isotope	3	293.1943	344	/	/	/
HI	pos	M299T291	Unknown	4	299.2121	291	x	x	x
HI	pos	M313T373	α-PEP Glycine Adduct	3	313.1912	373	x	x	x
HI	pos	M338T87	Oleamide	2	338.3420	87	x	x	x
PH	neg	M178T191	Unknown	4	178.0500	191	x	n.d.	n.d.
PH	neg	M289T48	Unknown	4	288.8766	48	x	x	x
PH	neg	M291T48	Unknown	4	290.8737	48	x	x	x
PH	neg	M295T25	Unknown	4	294.9531	25	x	x	n.d.
HI	neg	M178T336	Unknown	4	178.0500	336	x	n.d.	n.d.
HI	neg	M229T88	Unknown	4	229.0534	88	x	x	n.d.
HI	neg	M465T121	Cholesterol sulfate	2	465.3037	121	x	x	x

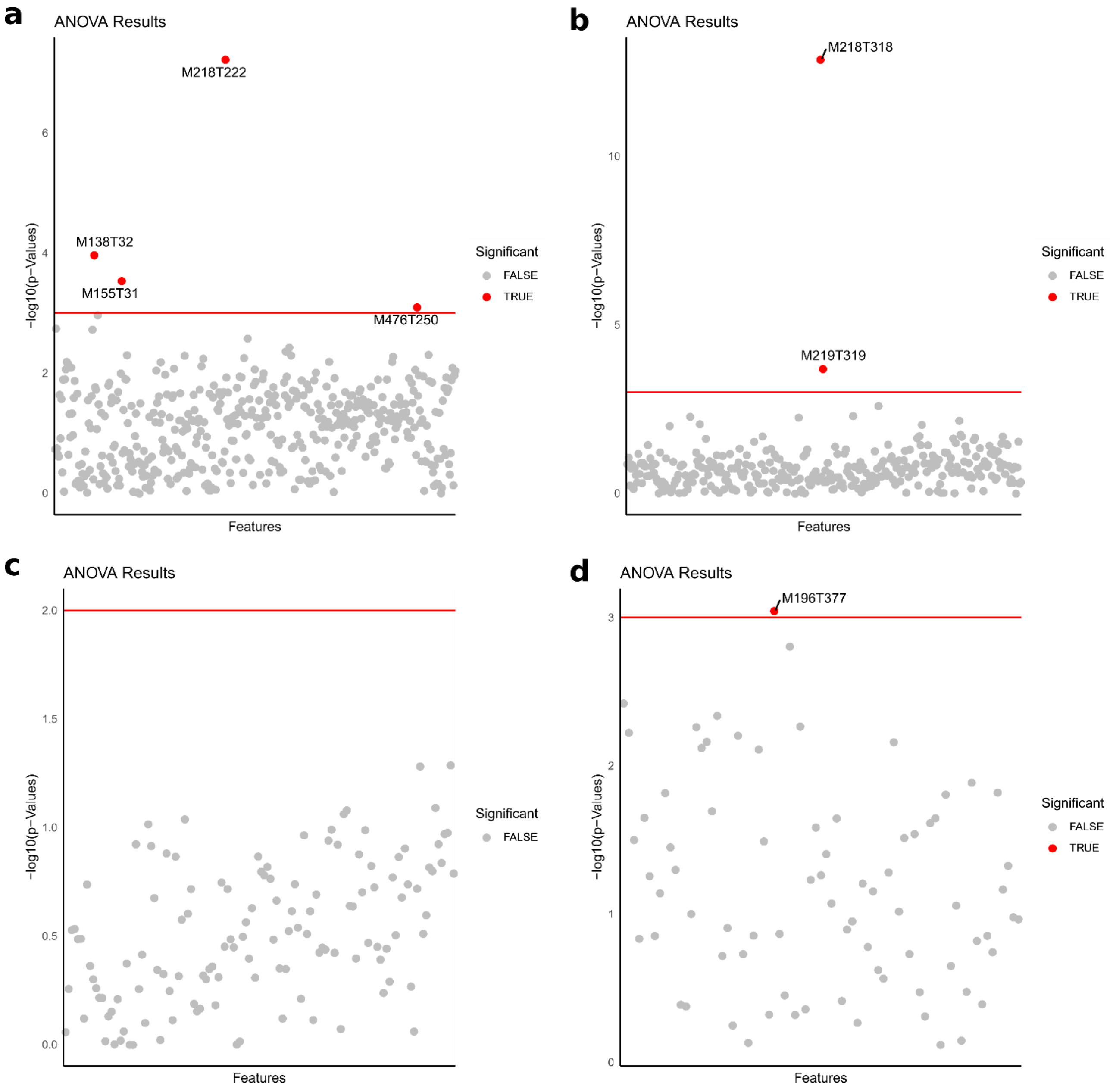

Figure S1 Results of one-way ANOVA for incubations using α-PBP. a = Extract 1 using PhenylHexyl column and positive mode; b = Extract 1 using HILIC column and positive mode, c = Extract 1 using PhenylHexyl column and negative mode, d = Extract 1 using HILIC and negative mode, e = Extract 2 using PhenylHexyl column and positive mode, f = Extract 2 using HILIC and positive mode, g = Extract 2 using PhenylHexyl and negative mode, h = Extract 2 using HILIC and negative mode.

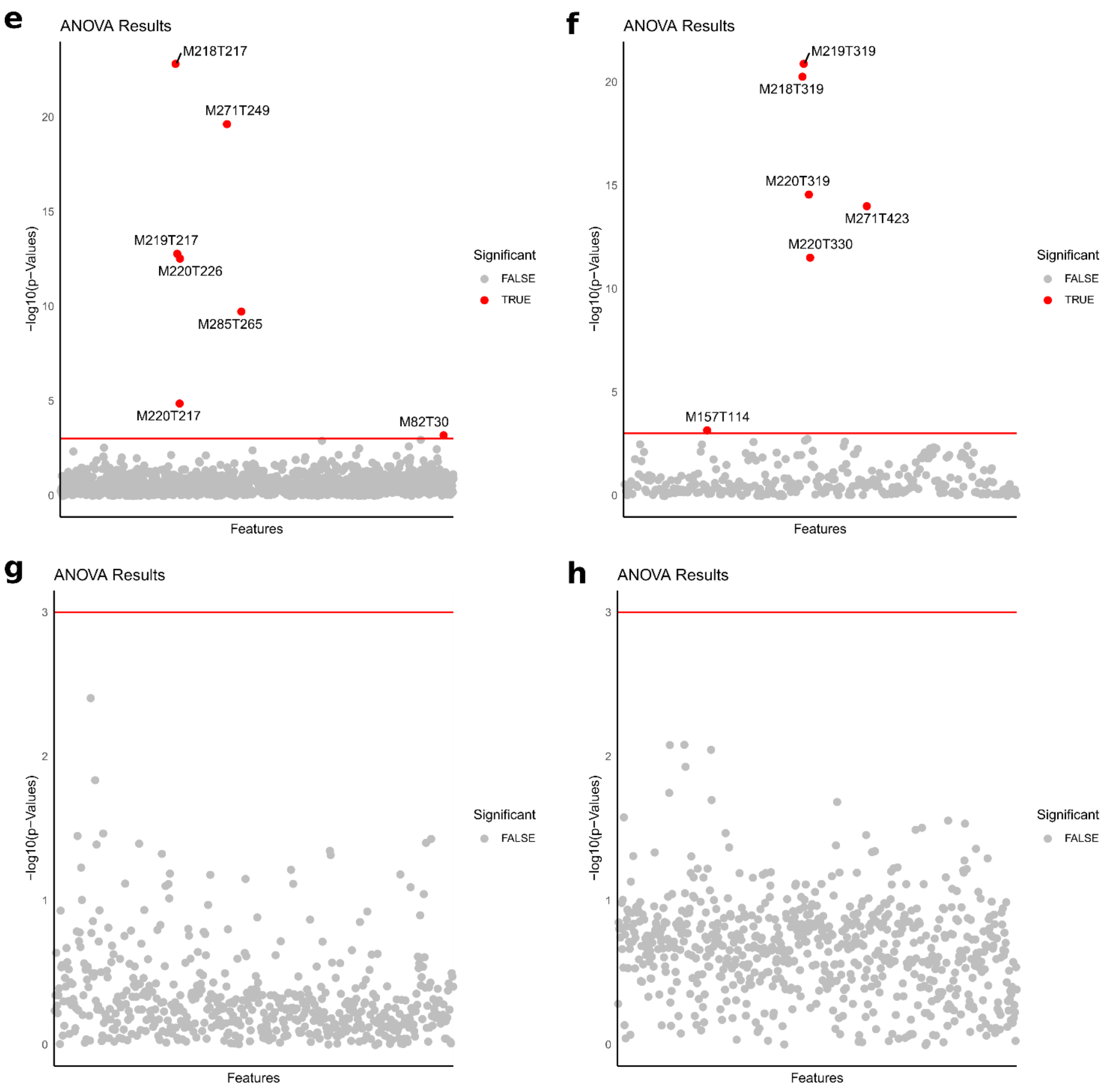

Figure S1 continued.

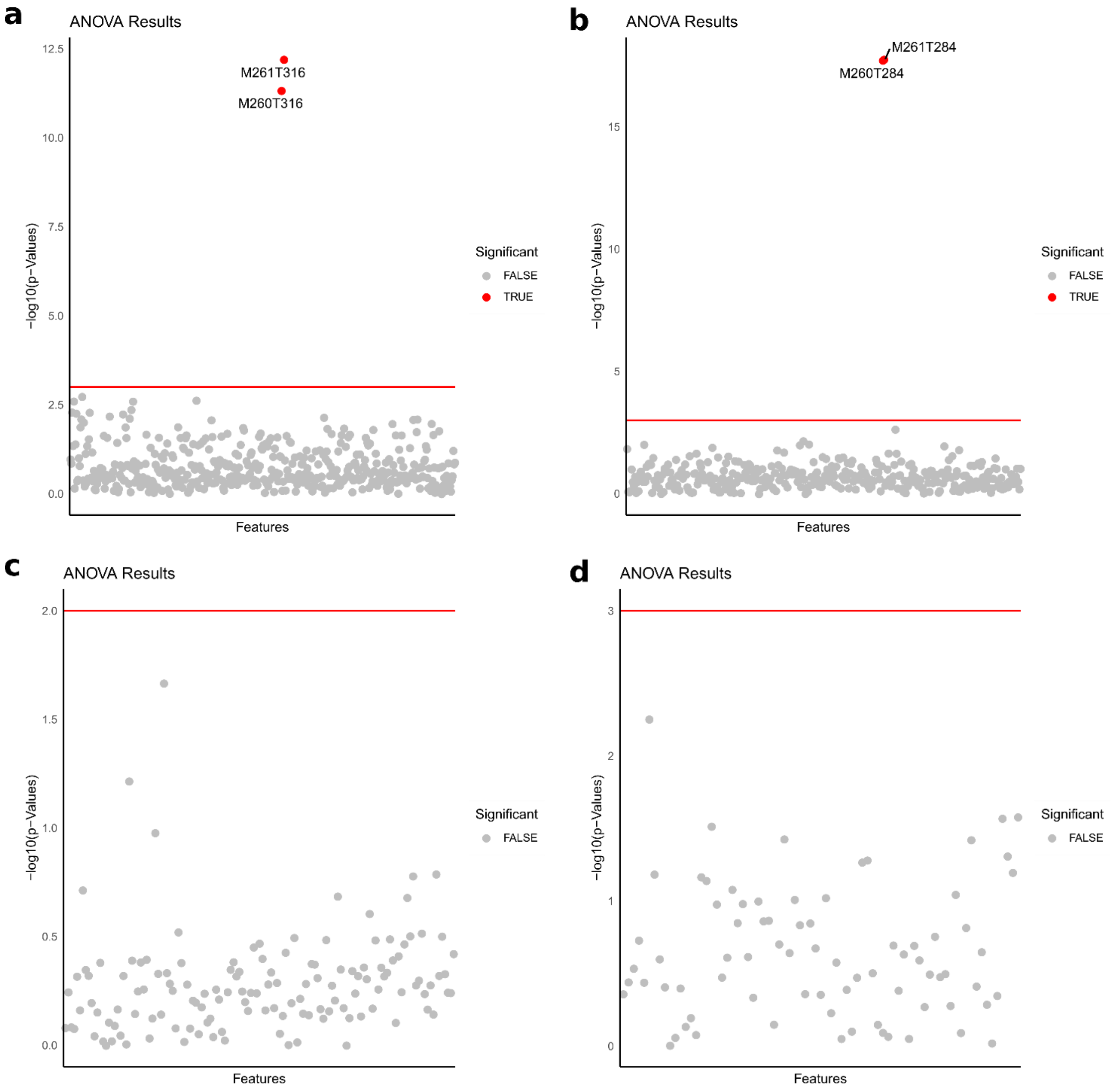

Figure S2 Results of one-way ANOVA for incubations using α-PEP. a = Extract 1 using PhenylHexyl column and positive mode; b = Extract 1 using HILIC column and positive mode, c = Extract 1 using PhenylHexyl column and negative mode, d = Extract 1 using HILIC and negative mode, e = Extract 2 using PhenylHexyl column and positive mode, f = Extract 2 using HILIC and positive mode, g = Extract 2 using PhenylHexyl and negative mode, h = Extract 2 using HILIC and negative mode.

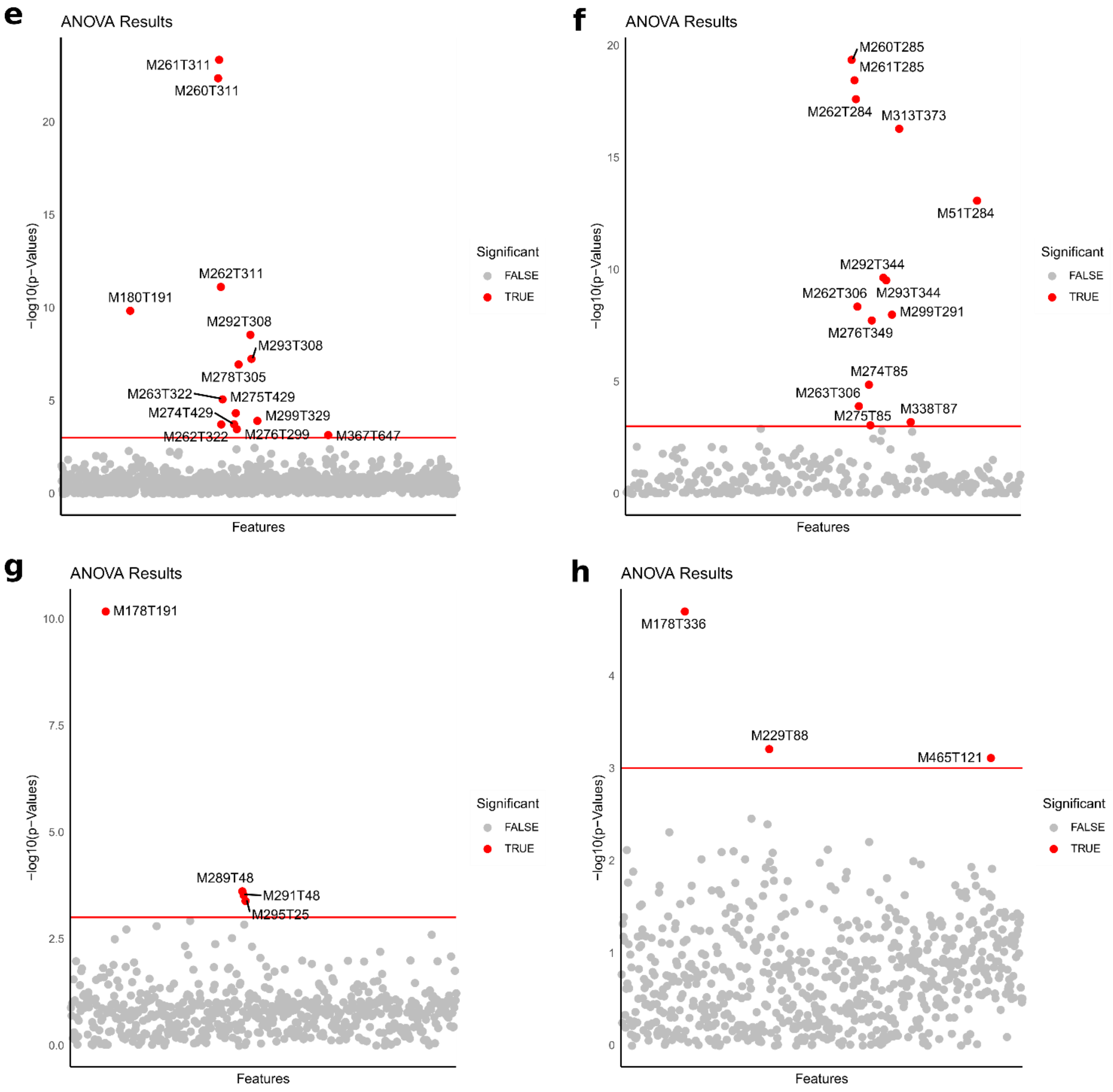

Figure S2 continued.

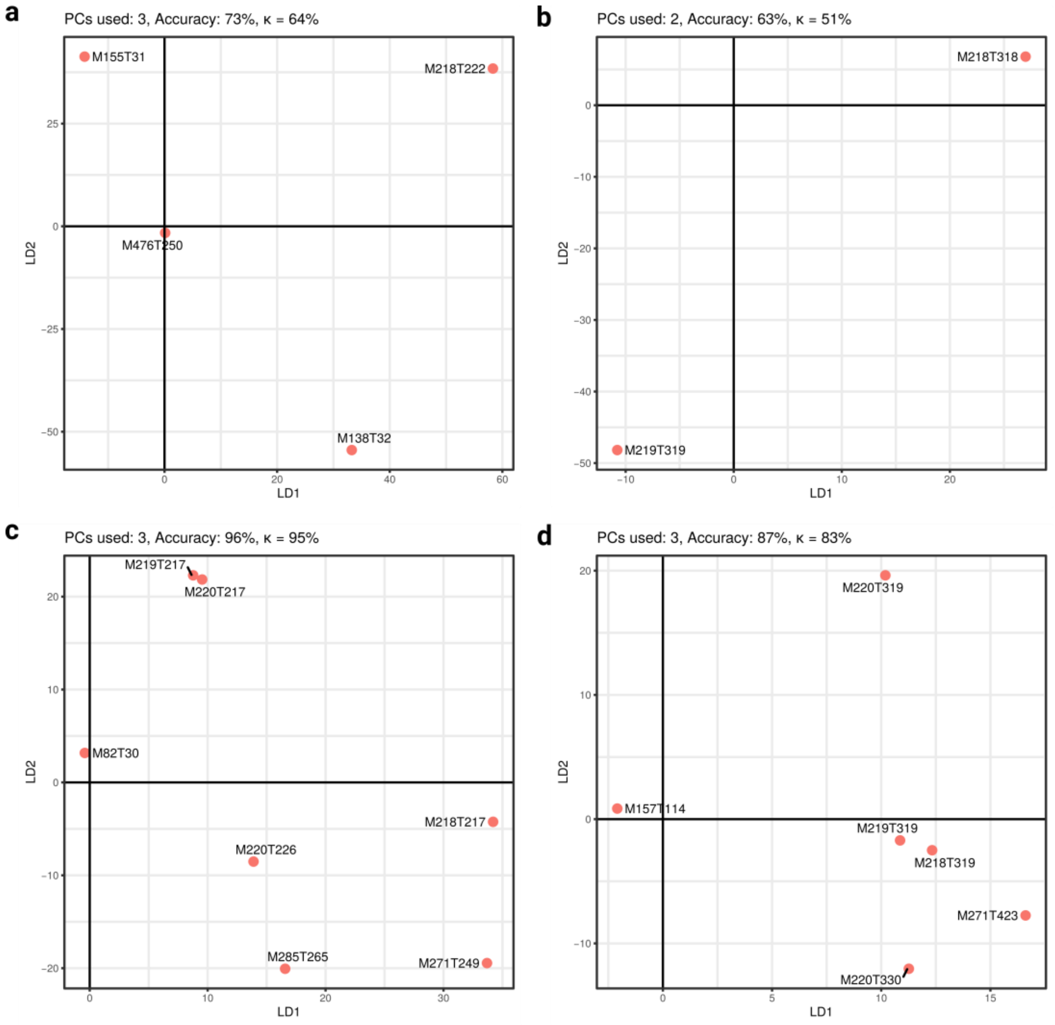

Figure S3 Loadings of PC-DFA for α-PBP with number of principal components used for discriminant function analysis, as well as prediction accuracy and Cohen's κ. a = Extract 1 using PhenylHexyl column and positive mode; b = Extract 1 using HILIC column and positive mode, c = Extract 2 using PhenylHexyl column and positive mode, d = Extract 2 using HILIC and positive mode, PC = Principal Component, LD = Linear Discriminant.

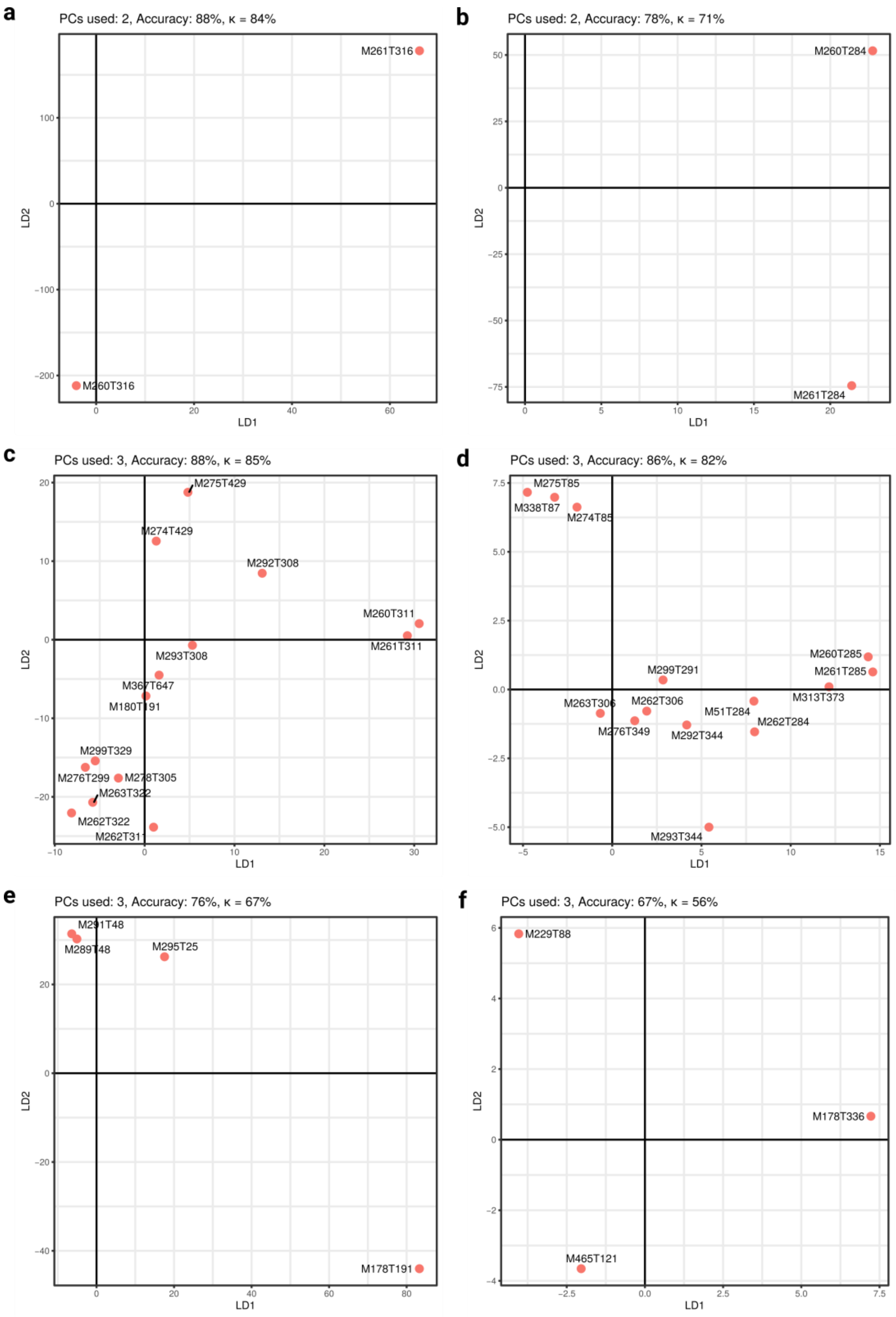

Figure S4 Loadings of PC-DFA for α-PEP with number of principal components used for discriminant function analysis, as well as prediction accuracy and Cohen's κ. a = Extract 1 using PhenylHexyl column and positive mode; b = Extract 1 using HILIC column and positive mode, c = Extract 2 using PhenylHexyl column and positive mode, d = Extract 2 using HILIC and positive mode, e = Extract 2 using PhenylHexyl column and negative mode, f = Extract 2 using HILIC and positive mode, PC = Principal Component, LD = Linear Discriminant.

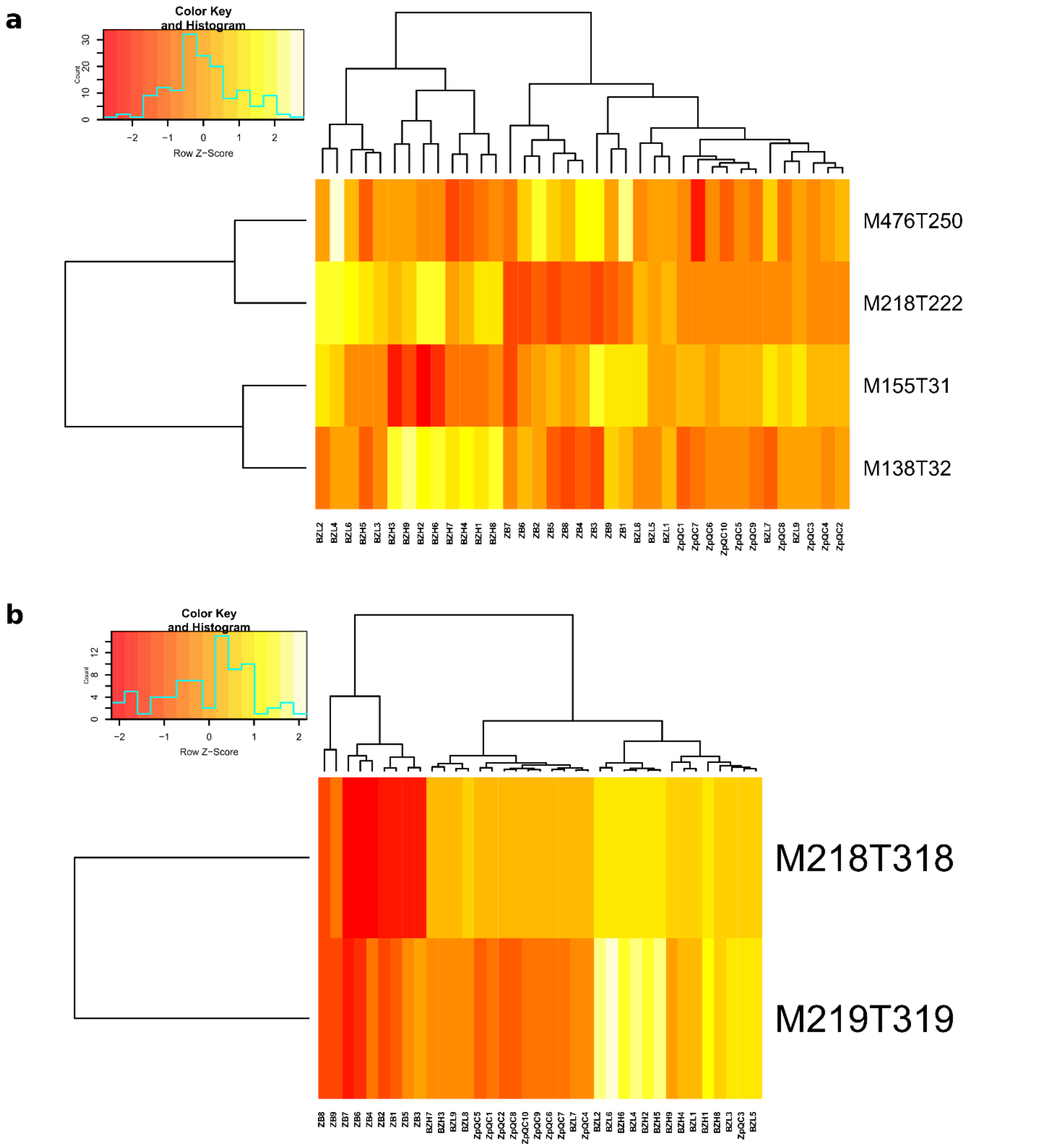

Figure S5 Heatmap after hierarchical clustering for α-PBP. a = Extract 1 using PhenylHexyl column and positive mode; b = Extract 1 using HILIC column and positive mode, c = Extract 2 using PhenylHexyl column and positive mode, d = Extract 2 using HILIC and positive mode.

c

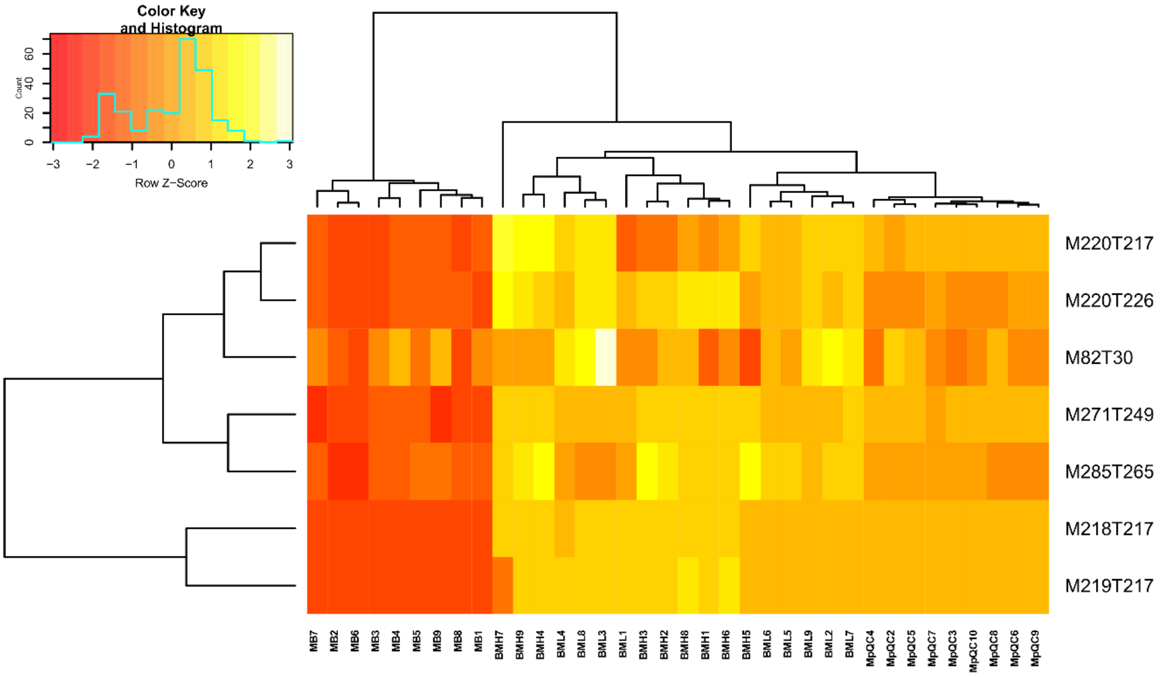

d

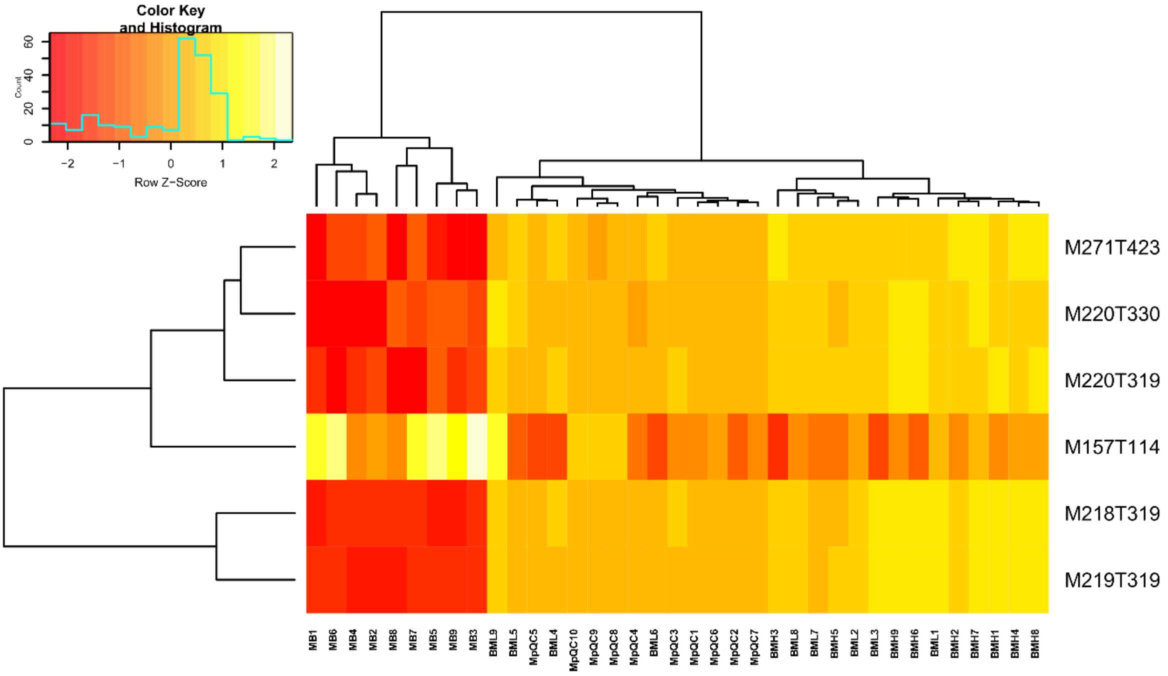

Figure S5 continued.

a

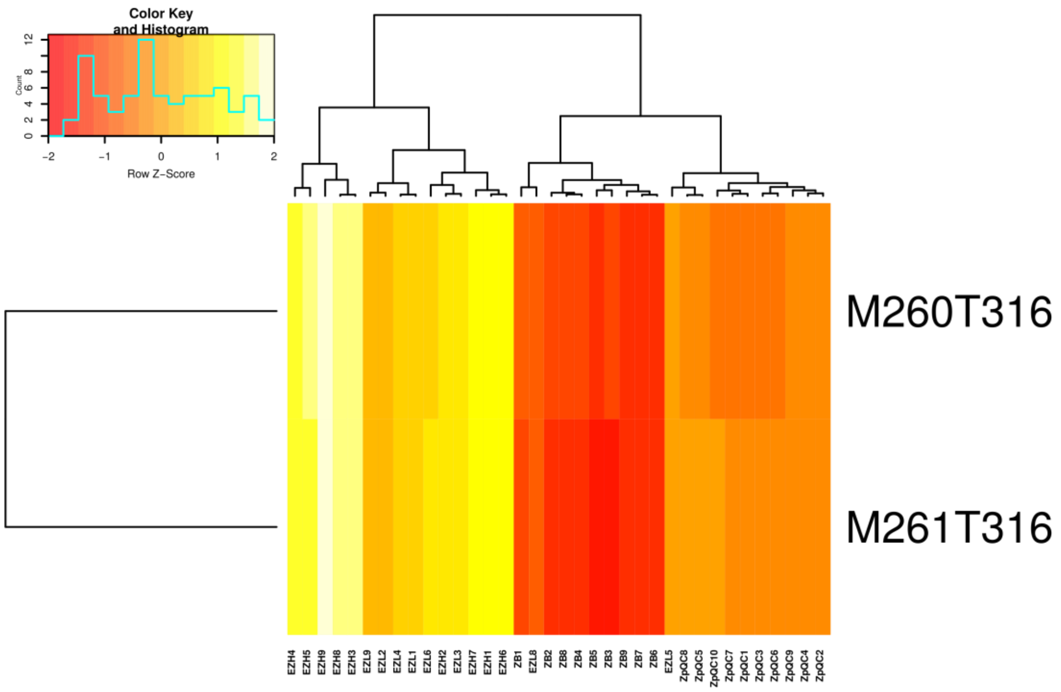

b

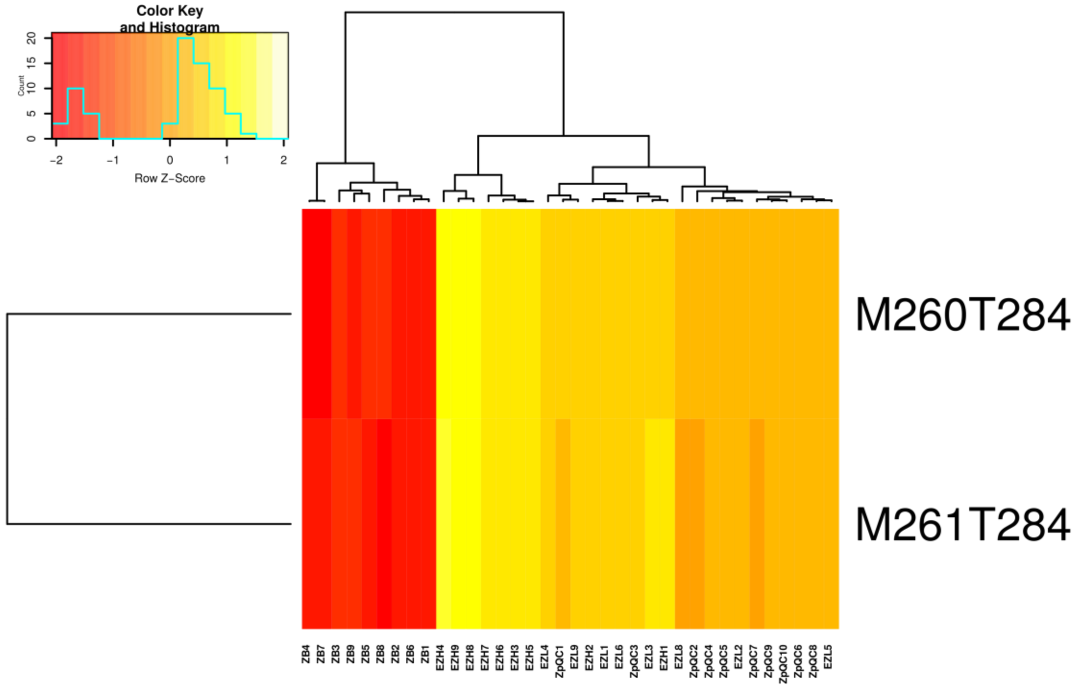

Figure S6 Heatmap after hierarchical clustering for α-PEP. a = Extract 1 using PhenylHexyl column and positive mode; b = Extract 1 using HILIC column and positive mode, c = Extract 2 using PhenylHexyl column and positive mode, d = Extract 2 using HILIC and positive mode, e = Extract 2 using PhenHex and negative mode, f = Extract 2 using HILIC and negative mode.

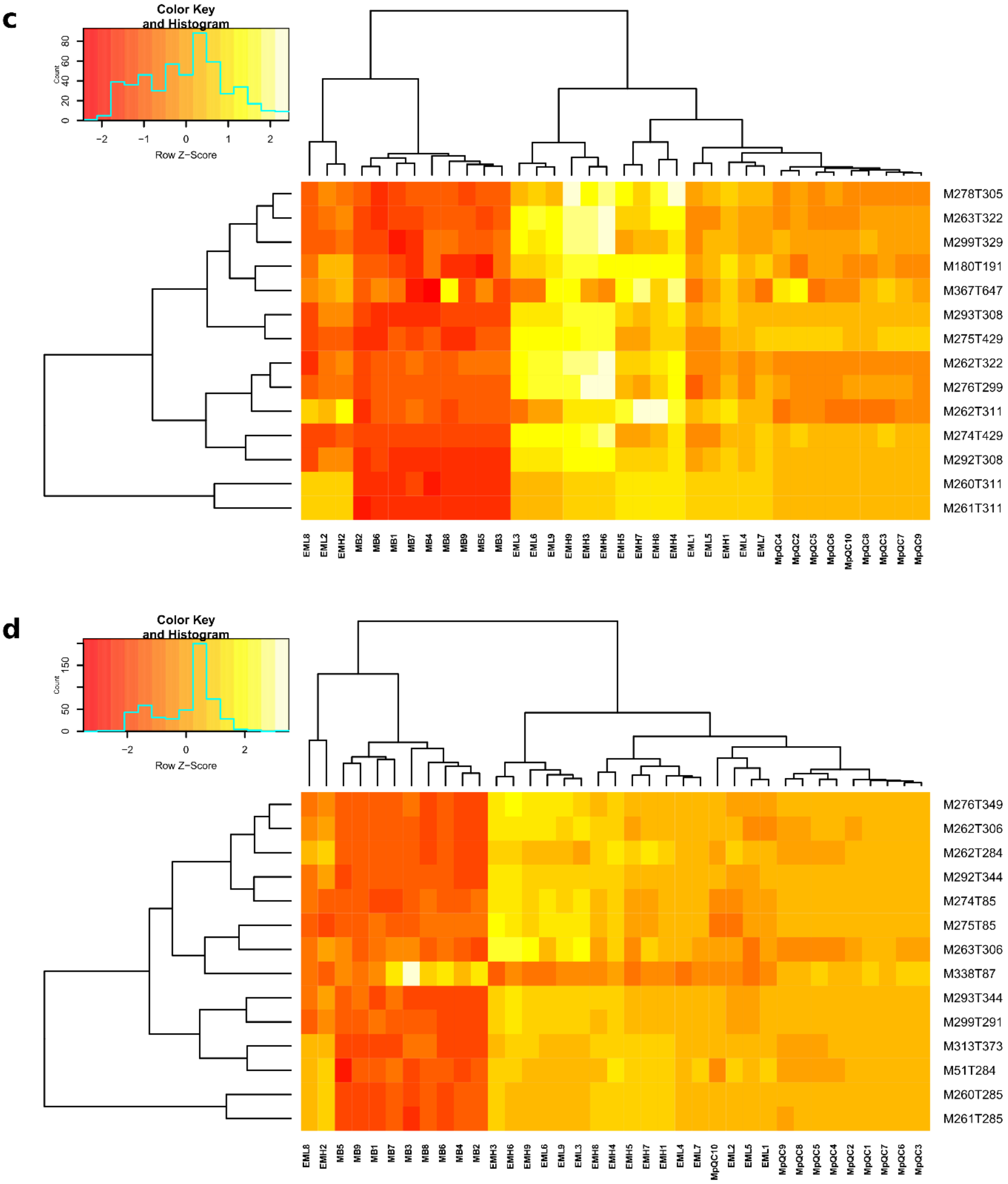

Figure S6 continued.

e

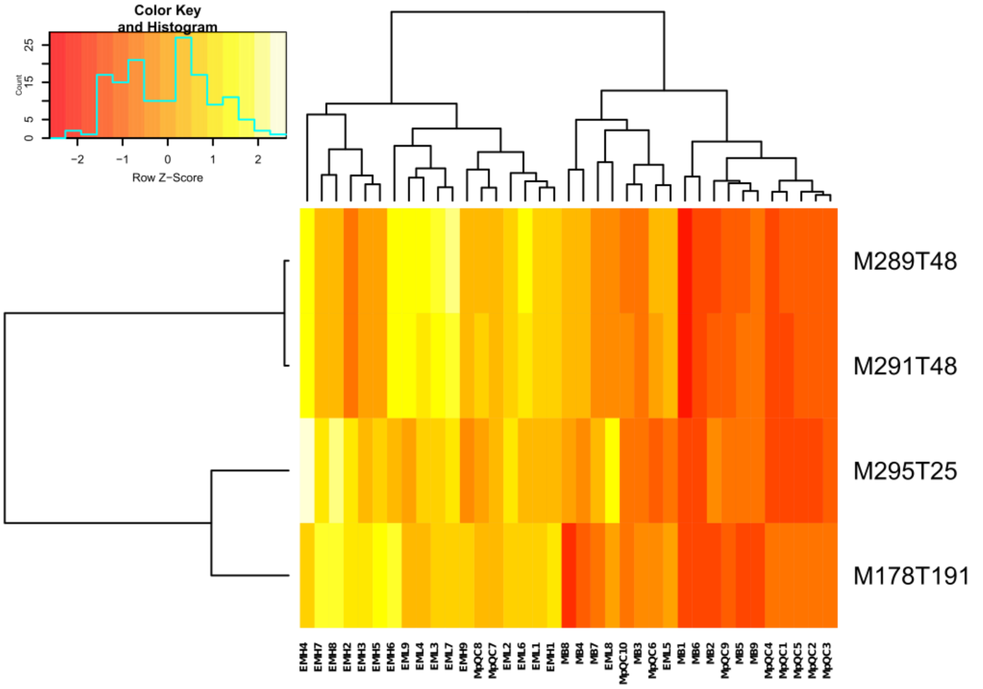

f

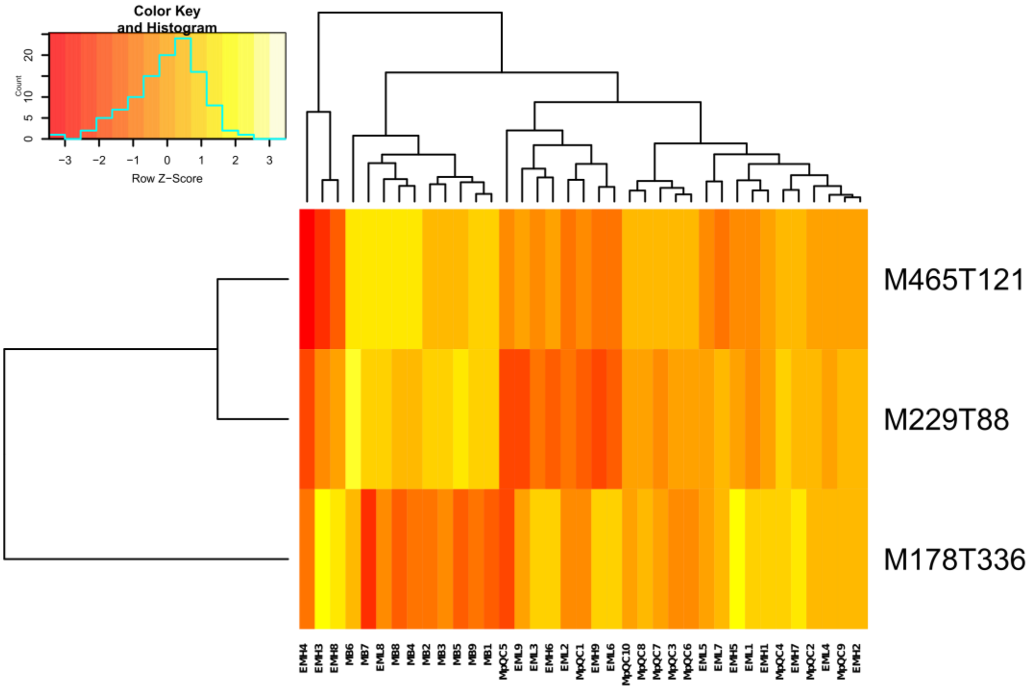

Figure S6 continued.
